# DNA-Polylactide Modified Biosensor for Electrochemical Determination of the DNA-Drugs and Aptamer-Aflatoxin M1 Interactions

**DOI:** 10.3390/s19224962

**Published:** 2019-11-14

**Authors:** Veronika Stepanova, Vladimir Smolko, Vladimir Gorbatchuk, Ivan Stoikov, Gennady Evtugyn, Tibor Hianik

**Affiliations:** 1 A.M. Butlerov’ Chemistry Institute of Kazan Federal University, 18 Kremlevskaya Street, 420008 Kazan, Russian; veronika1287@yandex.ru (V.S.); vas1993@yandex.ru (V.S.); leongard87@mail.ru (V.G.); Ivan.Stoikov@mail.ru (I.S.); gennady.evtugyn@kpfu.ru (G.E.); 2 Department of Nuclear Physics and Biophysics, Comenius University, Mlynska dolina F1, 842 48 Bratislava, Slovakia

**Keywords:** oligolactides, electrochemical DNA sensor, DNA aptamer, aflatoxin M1, self-assembling, phenothiazine dyes

## Abstract

DNA sensors were assembled by consecutive deposition of thiacalix[4]arenes bearing oligolactic fragments, poly(ethylene imine), and DNA onto the glassy carbon electrode. The assembling of the layers was monitored with scanning electron microscopy, cyclic voltammetry and electrochemical impedance spectroscopy. The configuration of the thiacalix[4]arene core determined self-assembling of the polymeric species to the nano/micro particles with a size of 70–350 nm. Depending on the granulation, the coatings show the accumulation of a variety of DNA quantities, charges, and internal pore volumes. These parameters were used to optimize the DNA sensors based on these coatings. Thus, doxorubicin was determined to have limits of detection of 0.01 nM (*cone* configuration), 0.05 nM (*partial cone* configuration), and 0.10 nM (*1,3-alternate* configuration of the macrocycle core). Substitution of native DNA with aptamer specific to aflatoxin M1 resulted in the detection of the toxin in the range of 20 to 200 ng/L (limit of detection 5 ng/L). The aptasensor was tested in spiked milk samples and showed a recovery of 80 and 85% for 20 and 50 ng/L of the aflatoxin M1, respectively.

## 1. Introduction

The determination of low-molecular compounds able to interact with native DNA has found increasing attention in the past decade due to the benefits of its possible application, e.g., monitoring of antitumor drugs, searching for new cytostatic drugs, and detection of toxins with specific DNA antibodies [1]. The mechanism of the appropriate interactions involves intercalation of the DNA helix with small flat aromatic fragments of the molecules or specific binding of the analytes onto the sites on the surface of the DNA receptor. Additionally, intercalators induce deep DNA damage due to reactive oxygen species [2,3] and hydrolytic chain cleavage [4]. All of these processes disturb configuration of the DNA molecules and affect their flexibility and internal volume [5]. Strict regulation of genotoxic species, as well as the narrow distance between the toxic and therapeutic doses of cytostatic drugs, calls for the development of reliable sensors for sensitive detection of such analytes. Today, the formation of the complexes between DNA and low-molecular compounds is mainly monitored by specific changes in the optical characteristics of the reactants, e.g., red shift of the bands in the UV-spectrum of DNA [6], changes in circular dichroism of the complexes [7], or excitation/quenching of fluorescence [8]. Such methods are sensitive, but cannot be used for the development of portable sensors within the point-of-care trend of medical diagnostics and food safety policy.

In contrast to conventional spectroscopic techniques, electrochemical DNA sensors offer obvious advantages with respect to use outside the lab, e.g., their simple and inexpensive production protocol, fast response, high sensitivity toward target interactions, compatibility with conventional instrumentation, applicability to colored sample analysis, easy modification for the use in flow-through and semi-automatic mode [9].

The detection of low-molecular compounds able to bind to DNA is mainly performed with electrochemical DNA biosensors using the redox signal of the analyte or a redox probe added to the sample, which is altered due to accumulation on the electrode interface and/or changes in the DNA configuration [9,10,11,12]. For this purpose, direct current (DC) voltammetry or electrochemical impedance spectroscopy (EIS) are frequently used with ferricyanide ion as a diffusionally free redox probe [13,14,15,16]. Being negatively charged, they indicate target interactions by decreasing current caused due to repulsion from phosphate groups of the DNA skeleton. For the same reason, charge transfer resistance is increased together with the capacitance responding to higher charge separation on the electrode interface. Being universal, such a detection principle has some drawbacks related to the contribution of non-specific adsorption and compensation of the above-mentioned changes by other charged species present in the solution.

Polymers are often used in the assembly of biosensors [17,18] and electrochemical sensors [19,20]. Depending on the polymer structure and properties, they can mechanically support receptors and redox labels [20,21,22,23], participate in the ion-exchange reactions [24], or prevent electrode poisoning and biofouling [25]. Cellulose derivatives, gelatin and Nafion are mostly mentioned in electrochemical sensors assembly. Meanwhile, oligomeric and polymeric forms of lactic acid also demonstrate some advantages related to low cost, biocompatibility, mechanical and chemical durability, and simple synthesis. Polylactic acids were successfully applied for dispersion of nanomaterials like Au nanoparticles [26,27] and carbon nanotubes [28,29,30] and a part of polyelectrolyte complexes and hybrid polymeric materials for sensor applications [31,32,33].

Further progress in the sensor applications of polylactic acid-derived materials requires their functionalization and regulation of permeability for low-sized ions and neutral molecules. Previously, we have shown that thiacalix[4]arenes functionalized with oligolactic fragments at the lower rim made it possible to regulate the morphology and transport properties of the thin films deposited onto the glassy carbon electrode by variation of the configuration of the macrocyclic core [34]. In this work, thiacalix[4]arenes bearing oligolactic fragments of different size were applied for deposition of polyelectrolyte complexes and detection of phenothiazine and anthracycline preparations and determination of aflatoxin M1 with specific aptamer.

## 2. Materials and Methods

### 2.1. Reagents

Lactic acid monomer was obtained from Fluka, DNA from salmon sperm, Methylene blue (MB) and Methylene green (MG), doxorubicin, poly(ethylene imine) (average molar mass 10 kDa, PEI) from Sigma-Aldrich (Darmstadt, Germany). Aptamer for Aflatoxin M1 (AFM1) reported by Nguyen et al. [35] has the following structure: 5′-NH_2_-ACT GCT AGA GAT TTT CCA CAT-3′ and was synthesized by Eurogentec (Seraing, Belgium).

**Figure 1:**
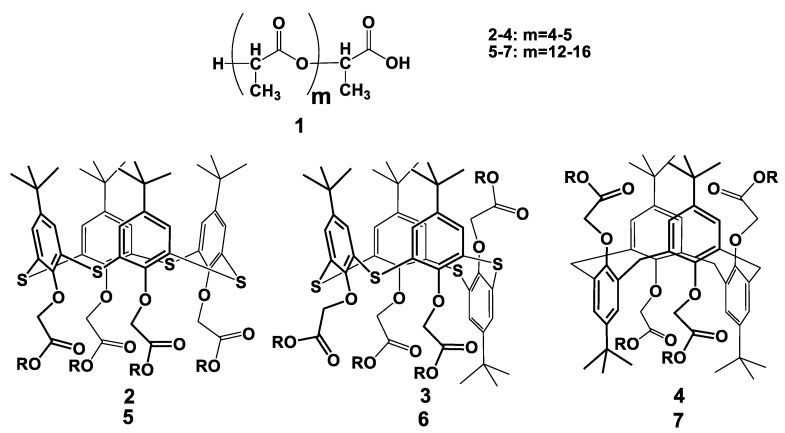
Chemical structures of oligolactide derivatives applied in the work.

The *p-tert*-butylthiacalix[4]arene derivatives bearing five residuals of lactic acid in the substituents **2**–**4** of the lower rim (Figure 1) were synthesized as described elsewhere [34]. Briefly, the monomer was mixed with hydroxycarbonyl derivative of *p-tert*-butylthiacalix[4]arene in *cone*, *1,3-alternate* (*1,3-alt*) or *partial cone* (*paco*) configuration and the mixture was heated at 180 °C at 20–40 mbar for five hours. Macrocyclic derivatives with elongated substituents **5**–**7** (Figure 1) were obtained by consecutive addition lactic acid and Sn octoate as catalyst, heating and evaporation of water and excessive lactic acid [36]. The average length of the substituents was established by the NMR ^1^H and MALDI TOF mass spectrometry data [37]. Electrochemical measurements were performed in Britton-Robinson buffer containing 0.04 M H_3_PO_4_, 0.04 M H_3_BO_3_, 0.04 M CH_3_COOH and 0.05 M Na_2_SO_4_. Other reagents were of analytical grade and used without additional purification. Working solutions were prepared using Millipore® water (Millipore SAS, Molsheim, France).

### 2.2. Apparatus

Voltammetric measurements in DC mode were performed with potentiostat-galvanostat AUTOLAB PGSTAT 302N (Metrohm Autolab b.v., Utrecht, the Netherlands) at room temperature in non-thermostated 5 mL working cell. The GCE (0.0167 cm^2^) modified with polymers and DNA (aptamer) was utilized as working electrode, the Ag/AgCl/3 M KCl as reference electrode and Pt wire as a counter electrode. Cyclic voltammograms were recorded at the scan rate of 100 mV/s. The pH values of solutions were measured with the digital pH-meter “Ecotest 001” (Econix-Expert, Moscow, Russia).

Electrochemical impedance spectroscopy (EIS) measurements were performed with the FRA module of the AUTOLAB PGSTAT 302N (“Metrohm Autolab b.v.”, the Netherlands) in 5 mL cell in the presence of 0.01 M K_3_[Fe(CN)_6_] and 0.01 M K_4_[Fe(CN)_6_]. Impedance spectra were recorded in the frequency range from 10^5^ to 0.04 Hz at the half-sum of the peak potentials of ferri- and ferrocyanide ions (214 mV), which is normally considered as an assessment of their formal standard potential. EIS parameters were calculated using the supplier software (NOVA, “Metrohm Autolab b.v.”) with the Randles equivalent circuit *R(C[RW])*.

The SEM microimages were obtained with the high-resolution field emission scanning electron microscope Merlin™ (Carl Zeiss, Jena, Germany).

### 2.3. Surface Layer Assembly

The 1 μL aliquot containing oligolactide derivatives dissolved in ethanol was drop casted on the GCE and allowed drying for 20 min at room temperature. Then the electrode was rinsed with Millipore^®^ water and working buffer. 2 μL of the PEI solution (1 mg/mL) were distributed on the working surface of the electrode and left for 6 min. After that, the electrode was rinsed again with deionized water and dried to form a soft wet film. After that, the 2 μL of the DNA solution (0.1 mg/mL) or AFM1 aptamer (1.0 μM) were put on the surface and left to dry for 10–15 min. In some experiments, the DNA aliquot was first thermostated for 30 min. at 90 °C and then transferred to the ice bath for 5 min. After that, it was introduced in the surface layer as described above.

The aptamer solution was preliminary thermostated at 90 °C and then conditioned at 0 °C for the structure folding. Finally, the electrode was washed with deionized water and used for DC measurements.

### 2.4. Biosensor Signal Measurements

For determination of the DNA specific interactions, changes in the charge transfer resistance or peak currents measured in direct current mode in the presence of phenothiazine dyes were used. Aptamer-AFM1 measurement was performed by incubation of the biosensor in the AFM1 solution followed by measurement of the phenothiazine dye peak current or charge transfer resistance in the ferricyanide solution. In real sample assay, the whole caw milk was first spiked with a definite quantity of AFM1 and then thermostated at 40 °C. After that, methanol was added to 3:1 *v*/*v* ratio and sediments were separated by centrifugation. The supernatant was diluted to 1:10 *v*/*v* ratio with the PBS. The aptasensor was incubated for 60 min and then its signal was recorded as described above.

**Figure 2:**
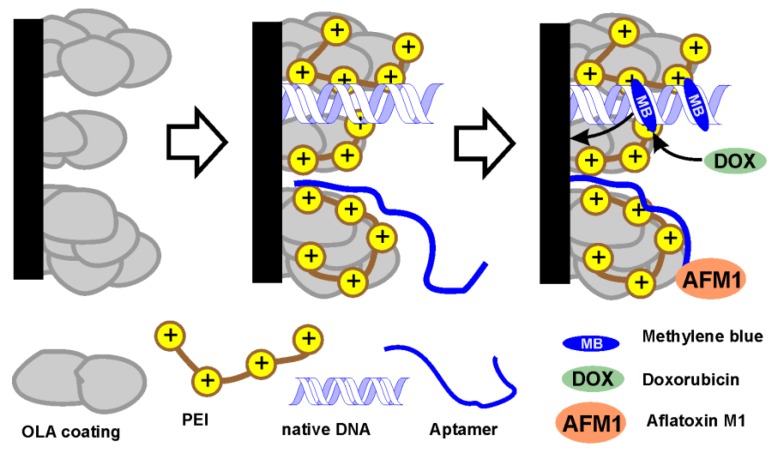
Schematic outline of the biosensor assembling and signal measurement.

Surface layer assembling and signal measurement are schematically presented in Figure 2.

The immobilization of DNA and aptamer is performed by consecutive loafing of positively charged PEI working as “molecular glue” and negatively charged biopolymers. For doxorubicin determination, the voltammetric signal of MB is used. The analyte suppresses the dye from the biopolymer and hence increases its signal. For AFM1 detection, the MG signal decreasing with increasing mycotoxin concentration is quantified. Details of mechanism are considered below in results discussion.

## 3. Results and Discussion

### 3.1. Surface Layer Characterization

Deposition of oligolactides **2**–**4** on the GCE has previously been investigated with SEM [34]. Here, long-chained derivatives **5**–**7** were deposited by drop-casting on the electrode interface and compared with each other.

**Figure 3:**
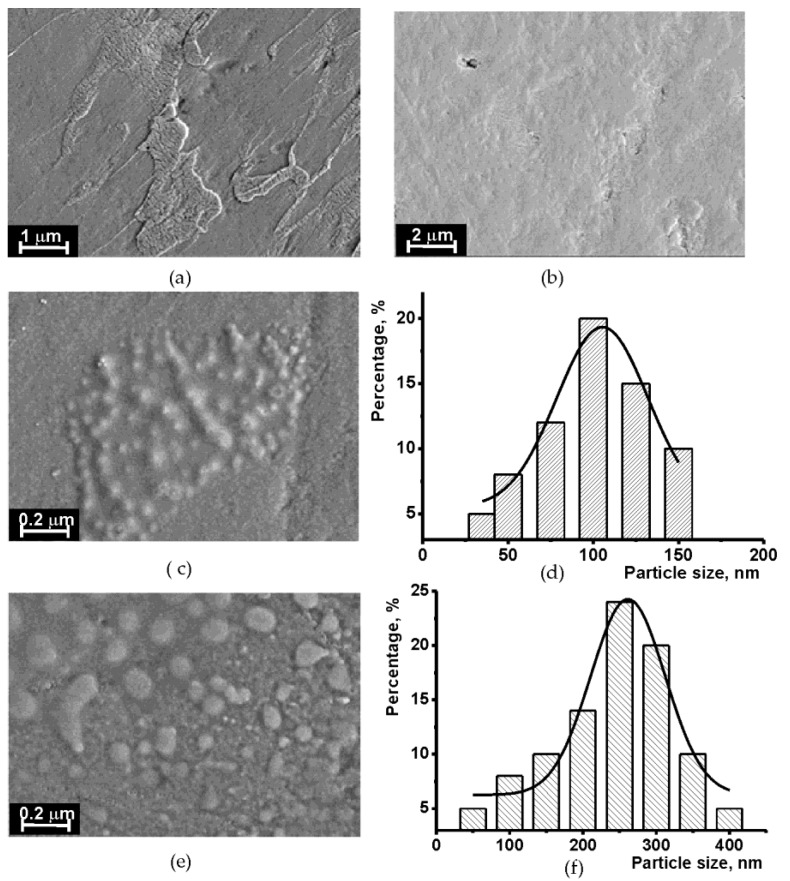
The SEM micro images (**a**–**e**) and domain size distribution obtained on bare GCE (**a**) and that covered with the 1.0 mg/mL OLA **1** (**b**), 0.1 mg/mL of OLA-*cone* **5** (**c**,**d**), OLA-*paco* **6** (**e**,**f**).

As in the case of the macrocyclic derivatives with shorter substituents, uniform dense films with regular distribution of roundish domains were formed in most films. This could be the result of the self-association of the macrocycles due to hydrophobic interactions between the aromatic rings of the core. The size of the domains varied from 70–120 nm for the OLA-*cone* **5** to 200–350 nm established for the *paco* **6** and the *1,3-alt* **7** configurations. As an example, the average size distribution determined from SEM images is presented in Figure 3 for *cone* **5** and the *paco* **7** configurations. In comparison with the derivatives with shorter substituents **2**–**4**, the ability to self-association of the compounds **5**–**7** was bigger. This resulted in the full coverage of the electrode surface reached at the higher loading of the polymer (1.0 mg/mL, aliquot 1 μL) against 0.1 mg/mL for the compounds **2**–**4**.

Previously [34], we confirmed regular deposition of oligolactides by EDS mapping performed with short-chain derivatives **2**–**4**. No significant changes in the element content for short- and long-chain derivatives were observed.

**Figure 4:**
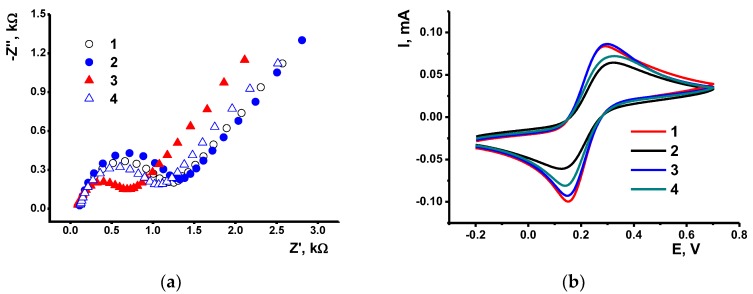
The Nyquist diagrams obtained on the GCE (1) covered with OLA-*cone* **5** (2), OLA-*paco* **6** (3) and OLA *1,3-alt* **7** (4) in the presence of 0.01 M K_3_[Fe(CN)_6_] and 0.01 M K_4_[Fe(CN)_6_] in 0.1 M KCl at 214 mV vs. Ag/AgCl. Frequency range 0.04–100 kHz, amplitude 5 mV (**a**) Cyclic voltammograms recorded in 0.1 M KCl at 100 mV/s on the GCE covered with the same layers. Polymer loading 1.0 mg/mL, aliquot 1 μL (**b**).

The assembling of the surface layer was also monitored by EIS using [Fe(CN)_6_]^3−/4−^ redox indicator. Appropriate Nyquist diagrams are presented in Figure 4. Deposition of oligolactides increased charge transfer resistance of the electrode interface. The degree of such an influence agrees well with the granulation shown by SEM images. Thus, maximal effect was found for the OLA-*cone* **5** (*R_et_* = 1.25 kΩ) and a lower one for the OLA-*paco* **6** (*R_et_* = 1.12 kΩ) and *1,3-alt* **7** (*R_et_* = 0.98 kΩ). Rather small changes in permeability of the OLA films coincide well with modest changes in the ferricyanide peaks on cyclic voltammograms and can be attributed to a large pore volume and high electron exchange confirmed earlier for the derivatives **2**–**4** [34]. Changes in the EIS parameters caused by polymer deposition generally coincide with the relative shifts of the peak currents on the cyclic voltammograms of [Fe(CN)_6_]^3−/4−^ (Figure 4b). However, separation between the various configurations of the macrocylic core of the polymer based on peak currents seems impossible due to lower sensitivity of the constant-current voltammetry to the conditions of electron transfer against EIS parameters.

It should be mentioned that deposition of the OLA with no macrocycle highly increased the *R_et_* value due to formation of a dense film and electrostatic repulsion of negatively charged ferricyanide ions (not shown, see [34]). Thus, the use of the thiacalix[4]arene core and spatial separation of the substituents bearing lactide fragments resulted in increased permeability of the surface layer toward small ions due to self-assembling of the modifier molecules and formation of well-defined roundish particles on the electrode surface.

The direct deposition of the DNA molecules onto the OLA film was ineffective due to their electrostatic repulsion from terminal carboxylic groups of oligolactides. Both the EIS parameters and ferricyanide peak currents tend to drift to the values typical for the modified electrode prior to the DNA deposition. For this reason, it was proposed first to treat the OLA film with the PEI polymer bearing positive charge of protonated amino groups.

Incubation of the GCE in 1 mg/mL PEI solution sufficiently improved DNA adsorption. This resulted in about twofold increase of the ferricyanide reduction peak and fourfold increase of the *R_et_* value to about 7–8 kΩ (OLA-*cone* **5**). Such changes in the above parameters can be explained by different influence of PEI-DNA adsorption on the quantity and reactivity of redox indicator ions. Indeed, neutralization of the negative charge of the oligolactide film inside the film by PEI would increase the quantities of the redox indicator transferred to the electrode. Meanwhile, charge transfer resistance becomes higher due general decrease in the surface layer permeability caused by additional deposition of bulky non-conducting DNA molecules onto the lactide-PEI layer.

Variation of the incubation time from 6 to 30 min and in the PEI concentration from 2 to 10 mg/mL did not significantly affect the above parameters. However, the repeatability of the signals decreased with increased incubation and PEI concentration due uncontrollable desorption of the surfactant and biopolymer molecules weakly bonded to the oligolactide underlying film. The DNA deposition was then performed using the following parameters of the surface layer assembling: macrocycle with oligolactide derivatives 1.0 mg/mL, PEI 2 mg/mL (6 min. incubation), DNA from salmon sperm 0.1–10 mg/mL (aliquot 2 μL).

### 3.2. Phenothiazine Dye Signals

**Figure 5:**
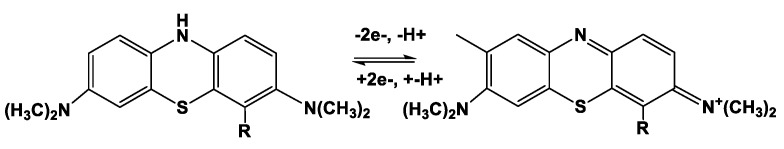
The scheme of redox behavior of Methylene blue (MB) or Methylene green (MG) (MB: R = H, MG: R = NO_2_).

**Figure 6:**
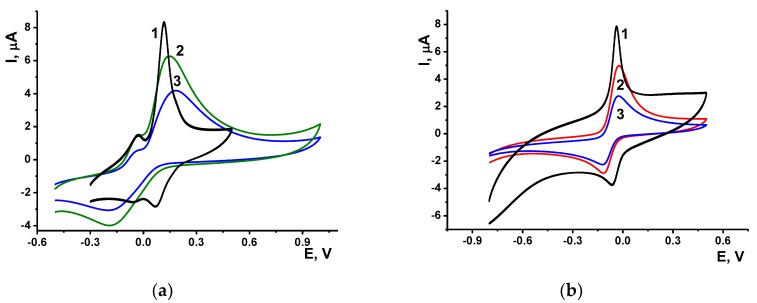
Cyclic voltammograms of 0.1 mM MG (**a**) and 0.1 mM MB (**b**), recorded on bare GCE (1), and that covered with OLA-*paco* **6**, PEI and native (2) and thermally denatured (3) DNA. Scan rate 100 mV/s, pH = 5.4.

Phenothiazine dyes MB and MG are often used as redox indicator of the DNA interactions. MB is able to intercalate DNA especially in the guanine reach regions [38] and meanwhile can be accumulated in minor grooves near adenine-cytosine reach part of the biopolymer [39]. For this reason, the signal of its electrochemical reduction consecutively decreased with the involvement of single-stranded DNA probe in hybridization with a target sequence [40]. Contrary to that, MG could not intercalate double stranded DNA because the nitro group at the aromatic system prevented penetration of the DNA helix [41]. On modified electrodes, both dyes exert quasi-reversible redox behavior (MB: R = H, MG: R = NO_2_, see Figure 5). This conclusion results from the voltammograms (Figure 6) containing a pair of well-defined peaks, which position and height depended on the GCE modification.

The peaks are better pronounced in weakly acidic media and insignificantly differ in the potentials with changes in the macrocycle modification. As example, the following peak potentials were obtained for OLA-*paco* **6** covered with PEI and DNA: *E_pa_* = 0.15 V for MG and −0.02 V for MB, *E_pc_* = −0.19 V for MG and −0.115 V for MB. For MG, the anodic peak is twice bigger than cathodic peak, and this ratio is about constant for all the thiacalix[4]arenes bearing oligolactic substituents.

The peak currents increased with the scan rate changed in the range from 5 to 500 mV/s. The dependence is linear in the plots of the peak current vs. square root from the scan rate. The slope obtained in logarithmic plots was found to be 0.78 (MG) and 0.84 (MB) for anodic peak current and 0.65 (MG) and 0.55 (MB) for cathodic peak currents indicating mixed diffusion-adsorption control of the electron transfer. In comparison with bare electrode (Figure 6, curve 1), deposition of oligolactides and DNA decreased the currents due to partial blocking of the electrode with electrochemical inactive substance. However, the peak shape remains the same.

For MG, pre-peaks were observed in some cases at about −0.05 V. They were attributed to the redox conversion of the dye adsorbed on the electrode. The MB peak is more symmetrical and narrower, indicating faster electron exchange in comparison with MG. In this particular case, intercalation of DNA does not affect the rate of electron exchange because the MB concentration added to the solution is high enough to compensate for any losses in DNA binding. Recording peak currents prior to and after the GCE modification indicated rather low resistance of the charge transfer carriers obtained earlier in EIS experiments. Deposition of 1.0 mg/mL of the compounds **5**–**7** resulted in decrease of the dye signals by 10–12% against bare GCE and improved repeatability of the peaks on voltammograms recorded in multiple potential scanning in the same dye solution. Thermal denaturation of DNA decreased the peaks of both MG and MB due to less regular configuration of the DNA and lower quantities of the dyes adsorbed on the DNA surface (Figure 6, lines 3). Meanwhile the shape of the curves and peak potentials were not affected by the DNA denaturation.

**Figure 7:**
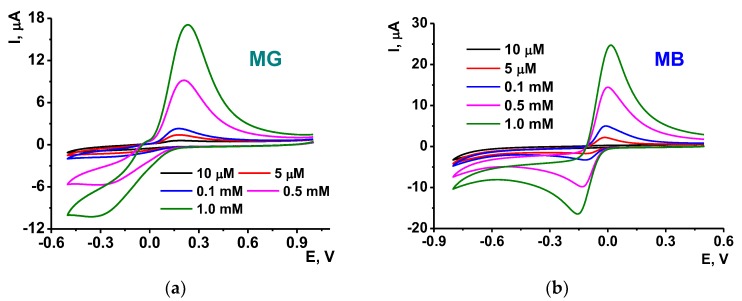
The cyclic voltammograms of various concentrations of the MG (**a**) and MB (**b**) recorded on the GCE covered with OLA-*cone* **5**, PEI and DNA. Measurements at scan rate 100 mV/s, pH = 5.4.

The adsorption of DNA did not prevent redox activity of both MG and MB probably due to their accumulation near the minor groove area of the DNA helix. In comparison with the GCE-OLA coating, the DNA adsorption increased the dye peak currents by 25–30%. Although MB could partially intercalate the DNA helix and hence loose its activity in redox reaction, presence of DNA did not affect the slope of appropriate calibration curves linearized in semi-logarithmic plots. As an example, Figure 7 presents the voltammograms of MG and MB recorded with different dye concentrations.

It should be mentioned that the conditions for the dyes transportation in the surface layer differ from those used in the works of Ferapontova et al. [42,43,44,45]. In them, DNA molecules are implemented in the hydrophobic self-assembled monolayer fully covering Au electrode. In such conditions, DNA intercalation with the MB provides long-distance electron transfer along the DNA helix. Here, DNA is located near the electrode in diffusionally spongy polymer layer and can regulated concentrations of diffusionally free redox probes but not prevent their access to the electrode interface. For this reason, the influence of the DNA-MB interaction of electrochemical properties of the biosensors is just opposite to each other.

The characteristics of their determination with different thiacalix[4]arene derivatives are summarized in Table 1. In them, anodic peak current was used as a signal of the dye oxidation measured at the reverse scan of cyclic voltammogram. In general, the dependence of the signal on the dye concentration is non-linear. For this reason, and because of the rather broad range of concentrations determined, empirical semi-logarithmic model was applied.

The comparison of the results obtained with thiacalix[4]arenes different in the length of the substituents (*cone* **2** vs. **5**, *paco* **3** vs. **6**, *1,3-alt* **4** vs. **7**) shows that long-chain substituents provide higher slope of the graph though the currents recorded are rather close to each other.

The parameters of the dye determination obtained on the electrodes covered with OLA that did not contain macrocycles are most similar, indicating the role of the core in the transfer reactions on the electrode. It should be mentioned that the metrological parameters of such calibration curves were also worse against other polymers tested (see *R*^2^ values and standard errors of the slope and intercepts). The comparison of the slopes of the calibrations with the SEM data indicating granulation of the coating it can be concluded that increased average size of particles formed from the polymer suspension on the step of drop-casting, the higher are the currents of dye oxidation. Similar consideration of reduction currents is complicated by worse reproducibility of the peak shape, which was in some cases converted to wave. Additionally, absolute reduction currents are significantly lower than anodic peak currents.

Similarity of the MG and MB behavior makes it possible to conclude that intercalation does not affect the signals recorded. Changes in the peak currents and calibration parameters might be due to different accumulation of the dyes on the coating and in the pores that compensate for the losses of the dyes bonded to DNA molecules. The range of concentrations determined is rather high in comparison with other electrochemical sensors including direct reduction of MB/MG on GCE.

### 3.3. Doxorubicin Determination

**Figure 8:**
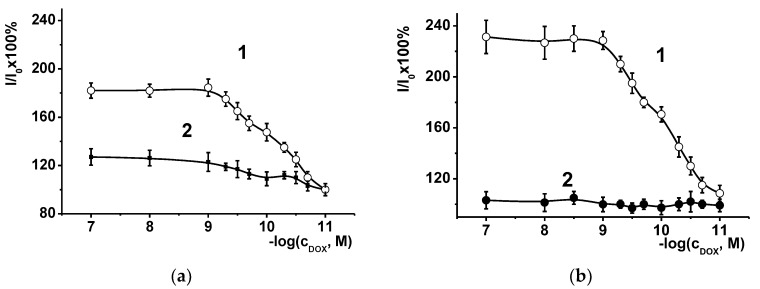
Relative increase of the anodic MB (1) and MG (2) peak currents recorded on the GCE with OLA-*cone* **2** (**a**) and **5** (**b**) polymers, PEI, and DNA after treatment with doxorubicin. Measurements at scan rate 100 mV/s, pH = 5.4. I_0_, μA—anodic peak current recorded prior to contact with doxorubicin; I, μA—the current recorded after 30 min incubation in doxorubicin, c_DOX_; M—doxorubicin concentration.

Doxorubicin belongs to the family of anthracycline antitumor drugs that are frequently used for the therapy of cancer due to their ability to prevent cancer cells fission [46]. After approval for medical application in 1974, it was criticized for rather high cardiotoxicity and a narrow gap between therapeutic and toxic dozen [47]. Recently we have shown that introduction of DNA in the redox active polymers can be used for sensitive determination of intercalating drugs by their influence on the redox activity of carrier [48]. Polyaniline electropolymerized in the presence of DNA changed its redox activity after treatment with doxorubicin. Moreover, preliminary treatment of the coating with the MB solution increased sensitivity of the drug determination. We checked this possibility with the electrode covered with polyelectrolyte complex. For this purpose, we treated the electrode covered with oligolactides, PEI and DNA first with the MB (MG), and then after washing with doxorubicin. Being electrochemically inactive in the potential area considered, doxorubicin did not alter the shape of the voltammetric peaks of the drugs, but dramatically affected the peak current. Figure 8 shows relative changes in anodic MB and MG peaks obtained for OLA-*cone* **2** and **5** derivatives.

Additional treatment with doxorubicin insignificantly altered the MG peak current, but sufficiently increased the MB peak. Such changes coincide well with those obtained for polyaniline–DNA composites saturated with phenothiazine dye. Probably, in both cases, doxorubicin competes for the same binding site as MB and provokes its release from the DNA helix. As a result, its redox activity, and hence the currents recorded, are increasing. As in the case of phenothiazine dye redox reactions, the use of the modifiers with loner substituents is preferable due to higher granulation and porosity of the layer and bigger amounts of DNA retained in the pores of the coating. This suggestion is indirectly supported by EIS measurements, where interaction with doxorubicin in the same measurement protocol decreased the charge transfer resistance twofold in the case of MB. It can be assumed that MB pushed from the DNA helix remains entrapped at the minor grooves and can be involved in electron exchange chain with ferricyanide redox probe as a diffusionally free electron acceptor.

The protocol proposed for doxorubicin determination involved 30 min incubation of the electrode in the 0.1 mM MB solution followed by twice washing in the buffer (pH = 5.4) and transfer into the doxorubicin solution for another 30 min. Shorter incubation periods resulted in lower current shifts and their higher deviation. It should be mentioned that absolute values of the currents could vary after the DNA deposition within 15–20% but relative current decay was reproducible with the standard deviation of 6–7% depending on the oligolactide used. Long chain macrocyclic derivatives provided higher slope of calibration curves (see Figure 8a vs. Figure 8b), so that the influence of macrocycle configuration was considered only for the compounds **5**–**7**. The results obtained are presented in Table 2. Limit of detection (LOD) was calculated from S/N = 3 criterion.

The sensitivity of doxorubicin determination is comparable with characteristics of other electrochemical sensors based on redox activity of polymeric matrix applied for DNA immobilization. Thus, electrostatic accumulation of doxorubicin on carbon nanotubes showed the LOD value of 2 nM [49], additional introduction of polylysine layer decreased the LOD to 1 nM [50]. Accumulation of double-stranded DNA on electropolymerized Neutral red resulted in detection of 0.1 nM doxorubicin by changes in the *R_et_* values in the presence of ferricyanide ions [51]. The use of DNA entrapped in the DNA decreased the LOD to 0.01 nM [52] and finally, competitive interaction with intercalated DNA allowed record LOD of 0.6 pM [48]. The advantages of the DNA sensor proposed cover higher stability of the surface layer which retains its response to doxorubicin within three months of storage in dry conditions.

Although selectivity of aptamer-AFM1 binding was proved on the step of aptamer selection, we have repeated the experiment with 1.0 nM AFM1 in the presence of tenfold excess of aflatoxin B1, a precursor of the AFM1 in metabolic conversion. No changes in the signal were observed for the aptasensor based on OLA-*cone* **5**. Meanwhile the response toward doxorubicin is sensitive to the antioxidants able to shift ratio of reduced and oxidized form of the dye and hence its values applied for the drug quantification. This influence found for 0.1–1.0 mM ascorbic acid can be eliminated by addition dilution of the sample prior to incubation.

### 3.4. Aflatoxin M1 Determination

Aflatoxins are secondary metabolites of fungi that exert immunosuppressive, mutagenic, teratogenic and carcinogenic effect. AFM1 is formed in living beings from aflatoxin B1. It is excreted in milk of lactating animals and is stable in the conditions of milk pasteurization and cheese ripening [53]. For this reason, the occurrence of the AFM1 is a serious problem of the food safety. The total aflatoxins level is limited in EU by 20 µg/kg in milk and 0.05 µg/kg in dairy products. In the United States, the FDA requirements 0.50 µg/kg AFM1 in milk [54]. To date, AFM1 has been determined by various HPLC techniques [55,56] and immunoassay [57]. Being sensitive, such methods are time and labor consuming, and cannot be used under field conditions. Electrochemical biosensors offer unique opportunities to preliminary control of foodstuffs to detect dangerous levels of AFM1 in a real time mode. Previously, we have shown that the use of specific aptamers together with macrocyclic labels based on thiacalix[4]arene platform provide the signal on the binding of AFM1 in direct voltammetry and EIS mode [35]. In both cases, ferricyanide redox probe was used and limitations in their transfer to the electrode increased with formation of aptamer-AFM1 binding at the electrode interface.

**Figure 9:**
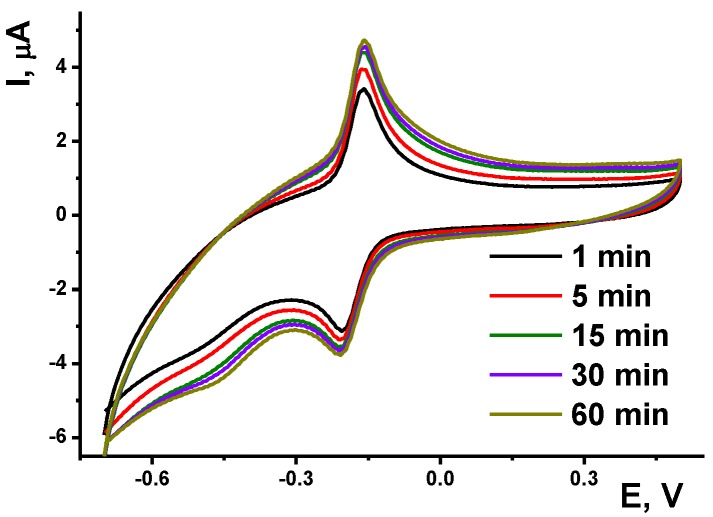
Dependence of the cyclic voltammograms of 0.1 mM MG solution recorded at the GCE covered with OLA-*1,3-alt* **7** and PEI on the incubation time in 1.0 μM aptamer specific to AFM1. Measurements at scan rate 100 mV/s, pH = 6.7.

In this case, aptamer to AFM1 was immobilized similarly to DNA by physical adsorption onto the OLA-PEI film. The deposition of the aptamer was monitored using the MG signal which is not influenced by the dye intercalation. Figure 9 shows changes in the MG peaks on voltammogram during the incubation of the electrode in 1.0 μM aptamer solution.

One could see, changes in the peak currents stopped to 30 min incubation. In a similar manner, other conditions for aptamer immobilization were optimized: buffer pH 6.7, aptamer concentration 1.0 μM.

Incubation of the aptasensor in the AFM1 solution resulted in partial decrease of the MG peaks due to limitation in the access of the dye to the binding sites of the aptamer. The effect regularly increased within 5–30 min. The anodic current was found to be more sensitive to the analyte addition.

The shift of the current linearly depended on the AFM1 concentration in the range from 20 to 200 ng/L (LOD 5 ng/L) with no respect of the configuration of the macrocycle bearing oligolactides. Appropriate dependency for OLA-*cone* **5** is expressed by the Equation (1) (n = 8, *R*^2^ = 0.9955).

Δ(I, µA) = (6.10 ± 0.05) − (0.020 ± 0.002) × (*c*_AFM1_, ng/L), n = 8, *R*^2^ = 0.9411 (1)

Application of *paco* derivative **6** decreased the slope of the curve to 0.017 µA/(ng/L) and that of *1,3-alt* **7** to 0.014 µA/(ng/L). Such changes are in agreement with the trends found for other properties of such coatings and DNA sensors assembled with such materials. Changes of the *R_et_* values recorded in EIS measurements performed in the presence of [Fe(CN)_6_]^3−/4−^ confirmed the role of AFM1 as a factor limiting access of the redox active dye to the aptamer. After the biosensor treatment, the charge transfer resistance increased due to lower permeability of the surface layer for redox probe and partial leaching of the positively changed MG molecules from the surface layer. Both impedimetric and voltammetry measurements show the same concentration intervals of the AFM1 determination but latter one is less reproducible (standard deviation for the signal to 100 ng/L equal to 7% for direct current voltammetry and 12% for EIS, five repetitions with single use sensors).

The LOD of the AFM1 achieved by voltammetry is sufficient for determination of the toxin on the level of its permissible concentrations in food products [54,55,56], and is comparable to characteristics of other electrochemical aptasensors reported. Thus, the use of intrinsic redox activity of polyaniline bearing covalently attached aptamer exerted LOD of 2 ng/L [35] and that with the thiolated aptamer directly attached to Au electrode 1 ng/mL (EIS measurements [58] DNA sensors with Neutral red covalently attached together with the aptamer to the polymeric support offered detection of 10 ng/L AFM1 in EIS mode [59].

The aptasensor developed has been tested in spiked milk samples containing 20 and 50 ng/L of AFM1. The sample treatment was adopted from [59] and involved removal of serum proteins by methanol treatment and ultracentrifugation. The dilution decreased the content of the solvent to 15% so that it could not affect the aptamer configuration and affinity to the analyte. The recovery of the AFM1 content determined from calibration curve obtained with standard solution in aqueous media with the electrode assembled on the OLA *cone* **5** was found to be 80 ± 5% for 20 ng/L and 85 ± 7% for 50 ng/L. Adsorption of interferences onto the pores of the polymer layers and desorption of MG molecules can be considered as one of the reasons of underestimation of the AFM1 content.

## 4. Conclusions

The modification of the thiacalix[4]arene with oligolactide fragments offers new possibilities for application of the materials in biosensor assembly. Simple synthesis, biocompatibility and variety of functional groups and interactions make it possible to construct various sensing systems that can be easily tuned for solution of particular analytical problems. The negative charge of terminal carboxylic groups does not allow direct immobilization of the DNA molecules but can be used for layer-by-layer assembling of polyelectrolyte complexes where DNA molecules preserve their native structure and ability to biochemical reactions. To monitor such reactions, it was proposed to use cyclic voltammetry of phenothiazine dyes, MG and MB. In reaction with DNA, they compensate for its negative charge and show reversible behavior on the electrode surface. The addition of doxorubicin results in increase of the MB signal due to substitution of the dye intercalated in the DNA helix. This increases dye concentration in the proximity of the electrode interface. MG, which does not intercalate native DNA, does not shift its signal in the presence of intercalator. This made it possible to detect sub-nanomolar concentration of doxorubicin. The application of the aptamer instead of DNA the surface layer of the biosensor made it possible to detect AFM1 specifically bonded to the aptamer. The reaction resulted in decrease of the voltammetric MG signal due to decre3asing permeability of the surface layer after aflatoxin M1 inclusion. The configuration of the macrocyclic core of the polymer influences the properties of the polymer layer and hence the charge of the interface and amount and reactivity of DNA/aptamer molecules adsorbed. This offers a simple and reliable way to control the performance of (bio)sensors by specifying the configuration of thiacalix[4]arene moiety and the length of oligolactide substituents. The electrochemical DNA- and aptasensors developed can find application in preliminary control of anticancer drugs and food contaminants.

## Figures and Tables

**Table 1:** Analytical characteristics of phenothiazine dyes determination with the GCE electrode modified with thiacalix[4]arenes bearing oligolactic fragments, PEI and DNA. I—anodic peak current, *c*—dye concentration. Average ± standard error calculated from five measurements, n—number of experimental points within linear piece of the curve.

OLA Macrocyclic Core	I, μA = a + b × log(*c*, M)				Concentration Range
	a	b	n	*R* ^2^	
	**MG**				
-	35 ± 3	4 ± 1	6	0.9055	10 μM–1.0 mM
*cone* **2**	43 ± 4	2.0 ± 0.2	6	0.9595	10 μM–1.0 mM
*paco* **3**	45 ± 4	3.2 ± 0.2	5	0.9337	10 μM–1.0 mM
*1,3-alt* **4**	48 ± 4	2.3 ± 0.1	5	0.9666	10 μM–1.0 mM
*cone* **5**	37 ± 3	7 ± 1	5	0.9211	10 μM–1.0 mM
*paco* **6**	52 ± 4	10 ± 1	5	0.9410	10 μM–1.0 mM
*1,3-alt* **7**	60 ± 6	9 ± 1	5	0.9324	10 μM–1.0 mM
	**MB**				
-	40 ± 4	5 ± 2	6	0.8922	10 μM–1.0 mM
*cone* **2**	35 ± 3	8 ± 1	6	0.9345	10 μM–1.0 mM
*paco* **3**	45 ± 5	7 ± 11	7	0.9751	10 μM–1.0 mM
*1,3-alt* **4**	60 ± 5	8 ± 1	7	0.9232	10 μM–1.0 mM
*cone* **5**	57 ± 4	12 ± 1	6	0.9445	10 μM–1.0 mM
*paco* **6**	55 ± 3	15 ± 2	6	0.9766	10 μM–1.0 mM
*1,3-alt* **7**	45 ± 3	13 ± 12	5	0.9701	10 μM–1.0 mM

**Table 2:** Sensitivity of doxorubicin determination with the GCE electrode modified with thiacalix[4]arenes bearing oligolactic fragments, PEI and DNA. Average ± standard error calculated from five measurements, n—number of experimental points within linear piece of the curve.

OLA Macrocyclic Core	Concentration Range, −log(C, M)	Slope, μA/log(C, M)	LOD, nM	*n*
*cone* **5**	9.0–10.5	−62 ± 2	0.01	9
*paco* **6**	7.0–9.0	−55 ± 2	0.05	7
*1,3-alt* **7**	7.0–9.5	−50 ± 1	0.10	7
